# Clinical and Genetic Features in 31 Serial Chinese Children With Gitelman Syndrome

**DOI:** 10.3389/fped.2021.544925

**Published:** 2021-04-29

**Authors:** Lingxia Zhang, Ke Huang, Shugang Wang, Haidong Fu, Jingjing Wang, Huijun Shen, Zhihong Lu, Junyi Chen, Yu Bao, Chunyue Feng, Guanping Dong, Jianhua Mao

**Affiliations:** ^1^Department of Nephrology, The Children's Hospital of Zhejiang University School of Medicine, Hangzhou, China; ^2^Department of Endocrinology, The Children's Hospital of Zhejiang University School of Medicine, Hangzhou, China; ^3^Chigene (Beijing) Translational Medical Research Center, Yizhuang, China

**Keywords:** Gitelman's syndrome, congenital tubulopathy, genotype, mutation-genetics, phenotype [mesh]

## Abstract

Gitelman syndrome (GS, OMIM 263800) is a genetic congenital tubulopathy associated with salt loss, which is characterized by hypokalemic metabolic toxicity, hypocalciuria, and hypomagnesemia. GS, which is typically detected in adolescence or adulthood, has long been considered a benign tubular lesion; however, the disease is associated with a significant decrease in the quality of life. In this study, we assessed the genotype–phenotype correlations based on the medical histories, clinical symptoms, laboratory test results, and whole-exome sequencing profiles from pediatric patients with GS. Between January 2014 and December 2020, all 31 consecutively enrolled patients complained of fatigue, salt craving, and muscle weakness. Sixteen patients demonstrated growth retardation, and five patients presented with nocturia and constipation. All patients presented with hypokalemic metabolic alkalosis, normal blood pressure, hyperaldosteronism, and a preserved glomerular filtration rate, and 24 of the 31 (77.4%) patients had hypomagnesemia. Homozygous, compound heterozygous, and heterozygous mutations in *SLC12A3* were detected in 4, 24, and 3 patients, respectively. GS patients often present with muscle weakness and fatigue caused by hypokalemia and hypomagnesemia. Therefore, early diagnosis of GS is important in young children to reduce the possibility of growth retardation, tetany, and seizures. Next-generation sequencing such as whole-exome or whole-genome sequencing provides a practical tool for the early diagnosis and improvement of GS prognosis. Further whole-genome sequencing is expected to reveal more variants in *SLC123A* among GS patients with single heterozygous mutations.

## Introduction

Gitelman syndrome (GS, OMIM 263800) is a genetic congenital tubulopathy associated with salt loss that is caused by defects in the sodium chloride cotransporter (NCCT, encoded by *SLC12A3*) with an autosomal recessive inheritance pattern (1). Epidemiological studies have shown that GS is the most frequent inherited tubulopathy with an estimated prevalence ranging from 1:40,000 in Europeans to 10.3:10,000 in the Japanese population (2), although the proportion of heterozygote carriers can reach up to 1% (3). However, the incidence of GS in the Chinese population is currently unclear (4).

The majority of GS patients present with mild and non-specific symptoms during adolescence or adulthood (1). The main characteristics of GS include hypokalemic metabolic toxicity, hypocalciuria, and hypomagnesemia. These electrolyte abnormalities lead to the common clinical manifestations of muscular weakness, salt craving tetany, and growth retardation. However, the phenotype of GS is highly variable and is linked to quality of life. Currently, a diagnosis of GS is based on clinical symptoms, biochemical abnormalities (i.e., normal/lower blood pressure, increased activity of the renin–angiotensin–aldosterone system, metabolic alkalosis, hypomagnesemia, and hypocalciuria), and genetic testing (5). Recently, next-generation sequencing (NGS) has begun to play an increasingly important role in the diagnosis of GS.

In the present study, we aimed to obtain a clearer picture of the genotype–phenotype relationships in Chinese patients with GS based on laboratory test data, clinical features, and medical histories, along with whole-exome sequencing profiles.

## Materials and Methods

### Study Subjects

Between January 2014 and December 2020, 31 probands (18 male and 13 female) with a clinical diagnosis of GS were recruited from the Children's Hospital of Zhejiang University School of Medicine. Patients were selected for inclusion in this study according to the classical GS standard: hyperaldosteronism, hypomagnesemia, hypocalciuria, renal hypokalemia, secondary hyperreninism, and metabolic alkalosis. However, plasma renin and aldosterone were not detected in every patient at diagnosis. In addition, hypomagnesemia or hypocalciuria was not described in the records of some genetically confirmed cases; therefore, we did not confirm all of the classical GS diagnostic criteria before genetic testing. Estimated glomerular filtration rates (eGFRs) were calculated using the Schwartz equation (6) that was developed for patients <18 years old. A total of 100 healthy children were selected as control subjects.

Patients with metastatic hypokalemia, potassium loss in the digestive tract, renal tubular acidosis, or medication history with cascara, diuretics, or ethanol were excluded from this study (7).

This study protocol was approved by the Ethics Committee of the Children's Hospital of Zhejiang University School of Medicine. All study participants or their guardians provided written informed consent before enrollment. The collected clinical data were statistically analyzed using an independent samples *t*-test.

### Laboratory Tests

An automatic biochemical analyzer was used to determine the blood concentration of electrolytes. A radioimmunoassay was used to determine plasma aldosterone, plasma renin activity, and plasma angiotensin.

### DNA Extraction

Genomic DNA was extracted from the peripheral blood of the patients using QIAamp Blood DNA Mini Kit (Qiagen, Milano, Italy) according to the manufacturer's instructions. DNA concentrations were determined using a NanoDrop spectrophotometer (Thermo Scientific, Waltham, MA, USA). The DNA samples were then stored at −20°C until analysis.

### Whole-Exome Sequencing

The whole-exome library was constructed using the Roche Nimble Gen Seq EZ Exome Enrichment Kit with capturing probes V2.0 (Roche, USA), and the DNA of total exons and their flanking introns were enriched. High-throughput sequencing was performed on an Illumina NovaSeq 6000 series sequencer (Illumina, USA), with at least 99% of target sequences sequenced at a 150 × reading depth.

### Data Processing and Annotation

Mutation analysis was performed as previously described (8, 9). For variant annotation and prediction of pathogenicity, the raw data were cleaned and then aligned to the National Center for Biotechnology Information human reference genome (hg18) using the BWA software package. GATK software was used to call single nucleotide polymorphisms and indels (<50 bp), and non-synonymous variations with a minor allele frequency <5% were screened using SIFT. All variations and related diseases were annotated using dbSNP, 1000 Genomes Project, ExAC, ESP, OMIM, Swiss-var, HGMD, ClinVar SNP, and/or disease databases. In addition, the Provean, SIFT, Polyphen2-HVAR, Polyphen2-HDIV, and Mutationtster software packages were used to predict functional changes in all detected variations, and MaxEntScan software was used to assess the impact of splice site variants. No significant difference was observed in the year at diagnosis, serum potassium, serum magnesium, blood pH value, epidermal growth factor receptor (eGFR), standard base excess, plasma renin activity, and plasma aldosterone concentration between patients under or above 7 years according to age of onset (Table 1).

**Table 1:** Fundamental data and laboratory findings in 31 consecutive children with Gitelman syndrome.

	**Gender**	**Year at onset**	**Year at diagnosis**	**eGFR (ml/min)**	**Serum potassium (mmol/L)**	**Serum magnesium (mmol/L)**	**Blood pH value**	**Standard base excess (mmol/l)**	**Plasma rennin activity (ng/ml h)**	**Plasma aldosterone concentration (ng/dl)**
1st	Male	9.1	13.1	101.9	2.2	0.62	7.494	8.6	5.09	174.57
2nd	Female	6.9	9.9	146.4	2.9	1.46	7.498	5.4	5.55	190.99
3rd	Male	13	13.9	130.6	2.6	0.5	7.469	6.6	–	–
4th	Female	5	10.9	112.9	2.46	0.5	7.439	4.1	4.43	131.51
5th	Female	3	5	112.8	2.6	0.68	7.427	4.2	4.57	162.42
6th	Male	4.5	4.6	122.5	2.3	1.23	7.44	1.8	2.8	174.22
7th	Male	4	4.1	104.5	2.65	0.82	7.45	4.4	7.73	421.94
8th	Female	8.7	8.8	141	2.5	0.51	7.471	2.7	3.1	137.9
9th	Female	11.6	11.7	120	2.8	1.46	7.429	3.1	5.3	221.36
10th	Male	6.5	9.5	98.5	2.4	0.55	7.49	4.8	1.22	–
11th	Female	9.25	9.3	103.1	2.1	0.56	7.424	6.9	–	–
12th	Male	4.7	4.7	101.1	2.5	0.65	7.459	4.7	2.65	361.4
13th	Female	11.9	11.9	122.7	2.4	0.52	7.459	5.7	5.5	327.47
14th	Male	9.5	9.7	122.2	2.5	0.69	7.459	2.3	3.09	129.34
15th	Female	11.7	11.8	141.8	2.5	0.46	7.485	5	0.55	102.42
16th	Male	12.8	12.1	120	2.6	0.6	7.466	7.2	6.9	152.66
17th	Female	9.8	9.8	107.1	1.95	0.59	7.462	4.2	5.45	179.12
18th	Female	5	14.8	120	2.3	0.64	7.442	5.6	21.24	198.46
19th	Male	8.8	8.8	120	2.1	0.65	7.458	4.6	5.4	335.03
20th	Male	11.9	11.9	120	1.9	0.65	7.478	3.5	3.17	483.8
21th	Male	4.6	5.6	90.8	2.3	0.5	7.515	3.9	4.11	162.32
22th	Male	11.8	11.8	120	2.4	0.57	7.414	5.4	6.4	140.1
23th	Male	13	13.9	112.5	2.3	–	7.467	0.6	22.4	308.78
24th	Female	10.6	10.6	108	2.3	0.66	7.494	5.5	3.55	117.68
25th	Male	12.5	12.6	108.7	2.3	0.48	7.367	3	3.53	164.46
26th	Male	6.9	6.9	93.9	1.8	0.47	7.526	4	–	241.31
27th	Male	12.1	12.1	92.1	2	0.83	7.55	−1.1	5.76	155.57
28th	Female	4.3	4.5	122.5	2.8	–	7.403	2.1	15.78	294.43
29th	Male	9.9	9.9	111.1	2.7	0.64	7.443	4.2	3.05	303.02
30th	Female	14.3	15.9	104.2	3	0.5	7.429	4.8	8.65	169.67
31th	Male	11.5	11.5	142.8	2.2	0.7	7.486	3.4	7.33	242.91
*N* = 11	Under 7Y at onset	5.04 ± 1.24	7.32 ± 3.48	111.45 ± 16.13	2.46 ± 0.3	0.75 ± 0.33	7.46 ± 0.04	4.09 ± 1.19	7.01 ± 6.44	233.9 ± 95.86
*N* = 20	Above 7Y at onset	11.19 ± 1.62	11.56 ± 1.85	117.49 ± 130.86	2.37 ± 0.29	0.64 ± 0.22	7.46 ± 0.04	4.31 ± 2.28	5.79 ± 4.57	213.66 ± 100.77
	*t*			−1.097	0.801	1.055	0.171	−0.296	0.583	0.518
	*P*			0.282	0.430	0.301	0.866	0.769	0.565	0.609

### Sanger Sequencing Validation

Sanger sequencing was used to confirm the candidate variations associated with GS. DNA extraction was performed as described above. Primer6 software was used for primer design, and polymerase chain reaction was performed using the QIAquick PCR Purification Kit (Qiagen, Milano, Italy). Following ABI BigDye sequencing protocols (cat. no. 4376484; Applied Biosystems; Thermo Fisher Scientific, Inc.), standard Sanger sequencing was performed on an ABI 3130XL genetic analyzer (Applied Biosystems; Thermo Fisher Scientific, Inc.). Sequencher DNA Sequence Analysis Software (http://www.genecodes.com) was used for data analysis.

## Results

### Characteristics of Patients With GS

Between January 2014 and December 2020, 31 consecutive patients (18 boys and 13 girls) with a clinical diagnosis of GS were recruited and sequenced from the Children's Hospital of Zhejiang University School of Medicine.

The clinical and biochemical features of the 31 patients with GS are shown in Table 1. All patients complained of fatigue, salt craving, and muscle weakness. Sixteen patients demonstrated growth retardation, and five patients presented with nocturia and constipation.

All patients presented with hypokalemic metabolic alkalosis, normal blood pressure, hyperaldosteronism, and a preserved glomerular filtration rate, and 24/31 (77.4%) patients had hypomagnesemia (normal range, 0.73–1.06 mmol/L). Twelve of the 31 patients presented with hypocalciuria. No significant difference was observed in the year of onset, year at diagnosis, serum potassium, serum magnesium, blood pH value, eGFR, standard base excess, plasma renin activity, and plasma aldosterone concentration according to age (Table 1).

### Mutations Identified in *SLC12A3*

A total of 36 different mutations were identified in 18 of the 26 exons of *SLC12A3* from the 31 consecutive patients, 25 of which were previously reported and 11 of which were novel (Supplementary Table 1).

Furthermore, among the 36 different mutations detected, the most common mutation types were missense mutations (23/36, 63.9%), splicing mutations (7/36, 19.4%), frameshift mutations (4/36, 11.1%), non-frameshift deletion (1/36, 2.8%), and non-sense mutation (1/36, 2.8%).

Among them, 21 mutation loci were located in the transmembrane coding region of the encoded protein NCCT, and 11 mutation loci were located in the coding region of the membrane.

**Figure 1:**
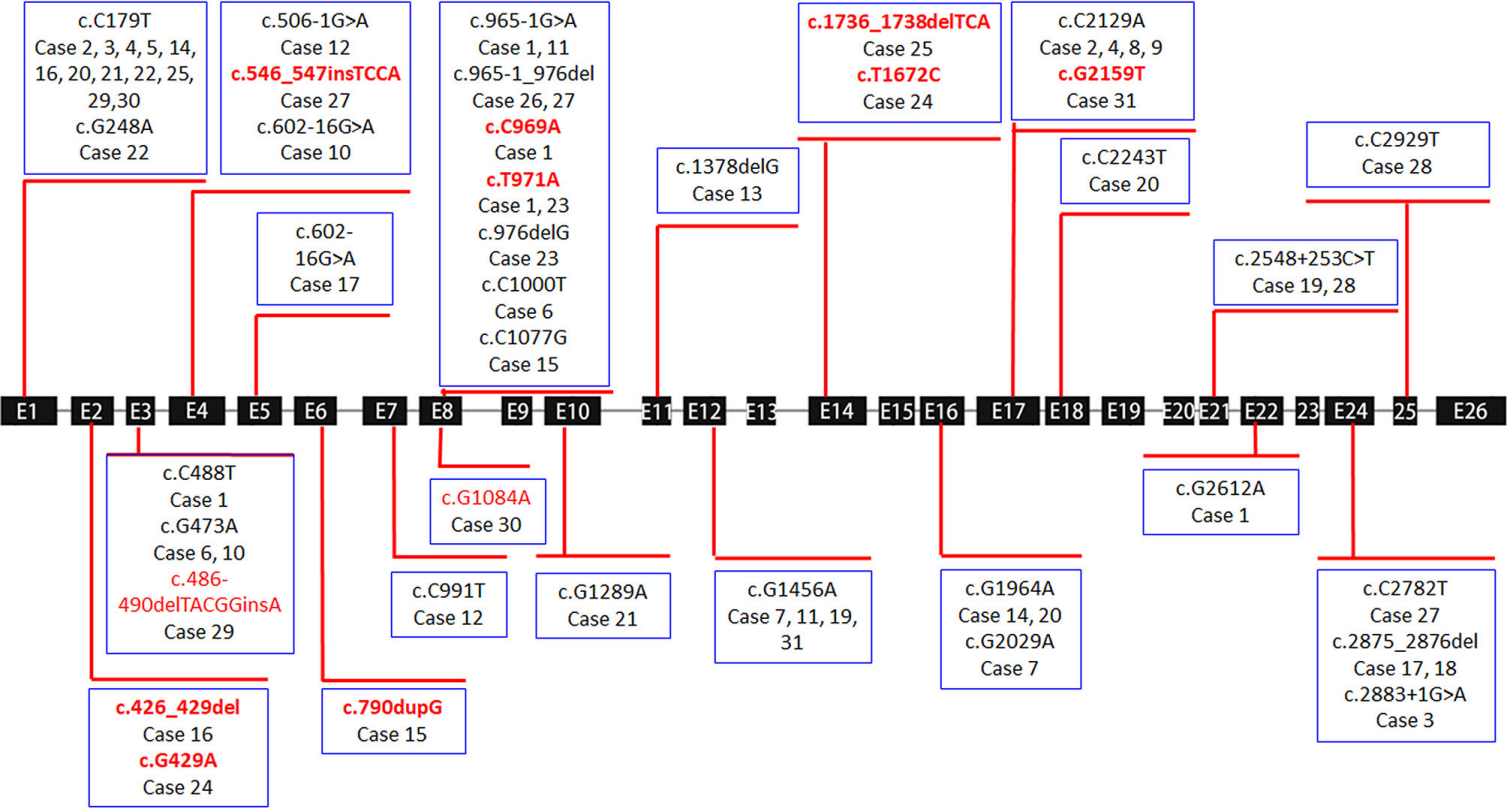
Exon structure of the *SLC12A3* gene with geometric shapes indicating the relative positions of different types of mutations. The 11 different types of novel mutations identified in this study are marked in red.

Only 3 of the 36 cases exhibited a single heterozygous mutation, which were all frameshift or splicing mutations (cases 13, 18, and 26). Homozygous mutations were found in four patients with non-sense or missense mutations (cases 5, 8, 9, and 28). All remaining children carried compound heterozygous mutations (24/31, 77.4%) (Table 2 and Figure 1).

**Table 2:** *SLC12A3* mutations by WES and corroborated sanger sequencing in patients with clinically diagnosed GS.

	**Location (Exon)**	**Variations**	**Protein change**	**Source of variation**	**ACMG evidence**	**Type of mutation**	**References**
1st	3	c.C488T^*^	p.T163M	Mother	Pathogenic	Missense	(10)
	22	c.G2612A^*^	p.R871H	Mother	Pathogenic	Missense	(11)
	8	c.965-1G>A^*^		Father	Pathogenic	Splicing	(12)
	8	c.C969A^*^	p.D323E	Father	Pathogenic	Missense	Novel
	8	c.T971A^*^	p.I324N	Father	Pathogenic	Missense	Novel
2nd	1	c.C179T^*^	p.T60M	Father	Pathogenic	Missense	(13)
	17	c.C2129A^*^	p.S710^*^	Mother	Pathogenic	Non-sense	(14)
3rd	24	c.2883+1G>A^*^		Father	Pathogenic	Splicing	(15)
	1	c.C179T^*^	p.T60M	Mother	Pathogenic	Missense	(13)
4th	1	c.C179T^*^	p.T60M	Father	Pathogenic	Missense	(13)
	17	c.C2129A^*^	p.S710^*^	Mother	Pathogenic	Non-sense	(14)
5th	1	c.C179T^**^	p.T60M	Mother	Pathogenic	Missense	(13)
6th	3	c.G473A^*^	p.R158Q	Father	Pathogenic	Missense	(10)
	8	c. C1000T^*^	p.R334W	Mother	Likely pathogenic	Missense	(16)
7th	12	c.G1456A^*^	p.D486N	Father	Likely pathogenic	Missense	(17)
	16	c.G2029A^*^	p.V677M	Mother	Likely pathogenic	Missense	(10)
8th	17	c.C2129A^**^	p.S710X		Pathogenic	Non-sense	(14)
9th	17	c.C2129A^**^	p.S710X		Pathogenic	Non-sense	(14)
10th	3	c.G473A^*^	p.R158Q	Father	Pathogenic	Missense	(10)
	Intron 4	c.602-16G>A^*^		Mother	Likely pathogenic	Splicing	(15)
11th	12	c.G1456A^*^	p.D486N	Father	Pathogenic	Missense	(17)
	8	c.965-1G>A^*^		Mother	Pathogenic	Splicing	(12)
12th	4	c.506-1G>A^*^		Father	Pathogenic	Splicing	(18)
	7	c.C991T^*^	p.T304M	Mother	Pathogenic	Missense	(19)
13th	11	c.1378delG^*^	p.G460Afs*32	Mother	Pathogenic	Frameshift	(20)
14th	1	c.C179T^*^	p.T60M	Mother	Pathogenic	Missense	(13)
	16	c.G1964A^*^	p.R655H	*De novo*	Pathogenic	Missense	(17)
15th	6	c.790dupG^*^	p.A264Gfs*47	Father	Pathogenic	Frameshift	Novel
	8	c.C1077G^*^	p.N359K	Mother	Pathogenic	Missense	(21)
16th	1	c.C179T^*^	p.T60M	Father	Likely pathogenic	Missense	(13)
	2	c.426_429del^*^	p.M143Ffs*10	Mother	Likely pathogenic	Frameshift	Novel
17th	5	c.602-16G>A^*^		Father	Pathogenic	Splicing	(15)
	24	c.2875_2876del^*^	p.R959Sfs*11	Mother	Pathogenic	Frameshift	(22)
18th	24	c.2875_2876del^*^	p.R959Sfs*11		Likely pathogenic	Frameshift	(22)
19th	12	c.G1456A^*^	p.D486N	Father	Pathogenic	Missense	(17)
	21	c.2548+253C>T^*^		Mother	Likely pathogenic	Splicing	(23)
20th	1	c.C179T^*^	p.T60M	Father	Likely pathogenic	Missense	(13)
	16	c.C1946T^*^	p.T649M	Mother	Likely pathogenic	Missense	(24)
	18	c.C2243T^*^	p.S748L	Mother	Likely pathogenic	Missense	(25)
21th	1	c.C179T^*^	p.T60M	Father	Pathogenic	Missense	(13)
	10	c.G1289A^*^	p.P430Y	Mother	Pathogenic	Missense	(1)
22th	1	c.C179T^*^	p.T60M	Mother	Likely pathogenic	Missense	(13)
	1	c.G248A^*^	p.R83Q	Father	Uncertain	Missense	(1)
23th	8	c.T971A^*^	p.I324N		Pathogenic	Missense	Novel
	8	c.976delG^*^	p.V326Sfs*44		Pathogenic	Frameshift	(26)
24th	2	c.G429A^*^	p.M143I	Father	Uncertain	Missense	Novel
	14	c.T1672C^*^	p.W558R	Mother	Uncertain	Missense	Novel
25th	1	c.C179T^*^	p.T60M	Father	Likely pathogenic	Missense	(13)
	14	c.1736_1738delTCA^*^	p.579_580del	Mother	Uncertain	Deletion	Novel
26th	8	c.965-1_976delGCGG ACATTTTTGinsACCGA AAATTTT^*^		Father	Pathogenic	Splicing	(20)
27th	4	c.546_547insTCCA^*^	p.T185Hfs*74	Father	Pathogenic	Frameshift	Novel
	8	c.965-1_976delGCGG ACATTTTTGinsACCGA AAATTTT^*^		Mother	Pathogenic	Splicing	(20)
	24	c.C2782T^*^	p.R928C	Father	Uncertain	Missense	(1)
28th	IVS 21	c.2548 + 253 C >T^**^		*De novo*	Likely pathogenic	Splicing	(23)
	25	c.C2929T ^*^	p.R977X	Father	Pathogenic	Non-sense	(17)
29th	1	c.C179T^*^	p.T60M	Mother	Likely pathogenic	Missense	(13)
	3	c.486-490delTACGGinA^*^	p.T163Rfs*7	Father	Likely pathogenic	Frameshift	Novel
30th	1	c.C179T^*^	p.T60M	Mother	Likely pathogenic	Missense	(13)
	8	c.G1084A^*^	p.G362S	Father	Likely pathogenic	Missense	Novel
31th	17	c.G2159T^*^	p.G720V	Father	Uncertain	Missense	Novel
	12	c.G1456A^*^	p.D486N	Mother	Pathogenic	Missense	(20)

* *Heterogeneous*.

** *Homozygous*.

*GS, Gitelman syndrome; WES, whole-exome sequencing*.

### Novel Mutations and Their Protein Structure Prediction

**Figure 2:**
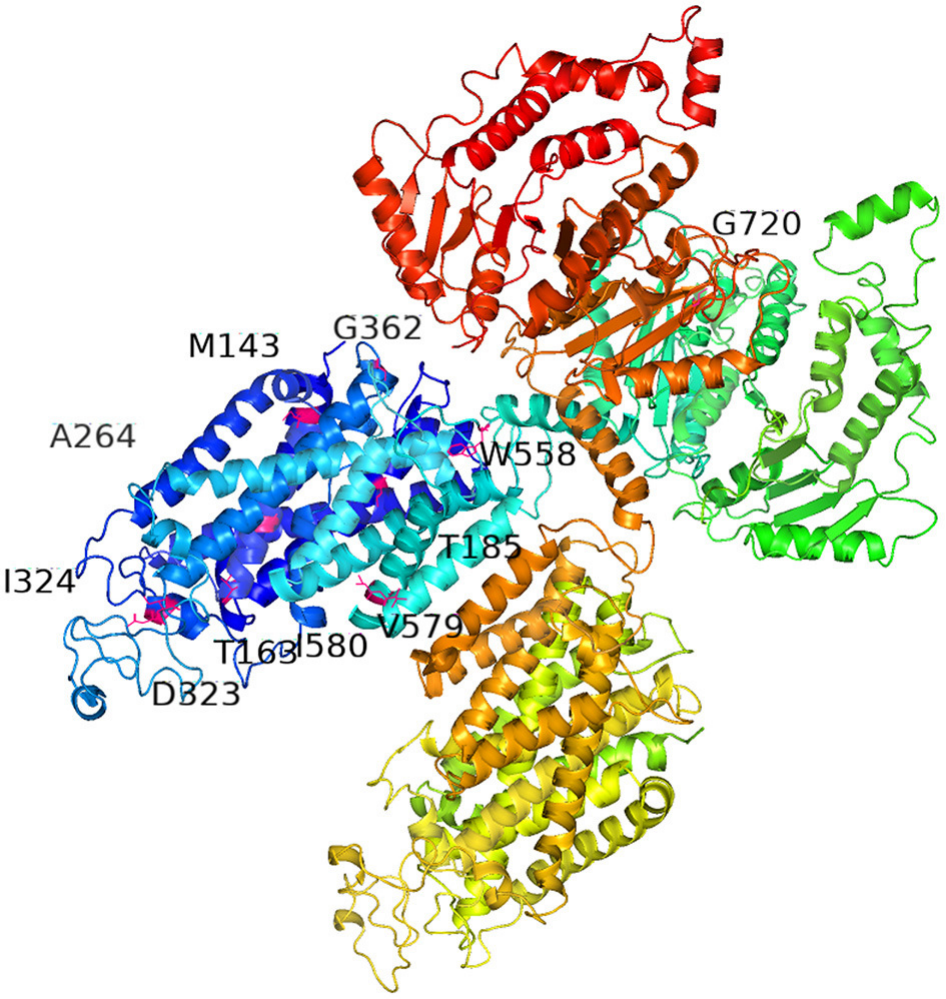
Three-dimensional model of sodium chloride cotransporter (NCCT) showing the locations of the 11 novel *SLC12A3* mutations, including missense, non-sense, deletion, and frameshift mutations, which resulted in the following amino acid variations: p.D323E, p.G362S, p.M143I, p.M143Ffs*10, p.W558R, p.579_580del, p.A264Gfs*47, p.T163Rfs*7, p.G720V, and p.T185Hfs*74 (hot pink), and p.I324N (light pink). These mutations are found in the amino acid permeases domain (amino acids 141–647; http://pfam.xfam.org/protein/P55017). Full protein sequences of the *SLC12A3* mutations were submitted to SWISS MODEL (http://swissmodel.expasy.org/) servers. The figure was generated using Pymol 2.3.2.

The predicted protein changes caused by the 11 novel mutations are summarized in Figure 2: p.D323E, p.G362S, p.M143I, p.M143Ffs*10, p.W558R, p.579_580del, p.A264Gfs*47, p.T163Rfs*7, p.G720V, and p.T185Hfs*74 are indicated in hot pink, and p.I324N is marked in light pink. These mutations were all found in the amino acid permeases domain (amino acids 141–647; http://pfam.xfam.org/protein/P55017) of NCCT protein.

### Genotype–Phenotype Relationships in Patients With 11 Novel Mutations

All patients harboring the novel mutations presented with hypokalemic metabolic alkalosis, hypomagnesemia, salt craving, and muscle weakness. Increased plasma renin appeared in some patients (cases 1, 16, 23, 25, 27, 30, and 32). Growth retardation was present in case 25, and tetany and hyperuricemia were found in case 30.

## Discussion

In this study, 31 children with newly diagnosed GS were subjected to clinical testing and genetic sequencing. All patients presented with hypokalemic metabolic alkalosis, normal blood pressure, hyperaldosteronism, and a preserved glomerular filtration rate, and 24/31 (77.4%) patients had hypomagnesemia. Whole-exome sequencing identified 4 cases with homozygous mutations, 3 cases with a single heterozygous mutation, and 24 cases with compound heterogeneous mutations in the causal gene *SLC12A3*.

GS is an autosomal recessive disorder caused by loss-of-function mutations in *SLC12A3* (24), and the majority of patients exhibit homozygous or compound heterozygous mutations of *SLC12A3*. However, based on *SLC12A3* mutation screening, 18–40% of GS patients are typically found to carry only one mutant allele (1, 14, 24, 27). Because GS is evidently recessive, and heterozygous relatives of patients with GS are clinically and metabolically asymptomatic, the reason for this phenomenon is unclear (28). Before the clinical introduction of NGS, the single-strand conformation polymorphism approach was most commonly used for mutation screening; however, this method is limited in resolution power. Moreover, traditionally, only exon and exon-boundary single nucleotide variants have been screened for GS, which may exclude relevant variations in the promoter region, introns, signaling poly(A) region, and both the 5′ and 3′ untranslated regions, which may explain why these patients were identified as single heterozygous carriers (24). In the present study, whole-exome sequencing and subsequent Sanger sequencing were applied in clinically diagnosed GS patients, revealing that only 3/31 (9.7%) carried heterozygous mutations (frameshift mutation in cases 13 and 18 and a splicing mutation in case 26). Thus, the incidence of heterozygous mutation in our study is much lower than that reported previously (18–40%) (1, 14, 24, 27). Although the reason for this discrepancy is unknown, we recommend whole-exome or whole-genome sequencing for patients with GS to identify undiscovered variations in *SLC12A3*, which may increase the identification rate for genetic diseases such as GS.

GS has long been deemed a benign disorder, which is generally diagnosed during adolescence or adulthood. However, with the introduction of NGS, the establishment of a GS diagnosis is becoming easier in young children. This can be meaningful as GS is associated with a significant reduction in quality of life and is not always a benign tubulopathy, as mentioned by Cruz et al. (16).

GS often manifests with symptoms of cramps/muscle weakness, dizziness, fatigue, salt craving, thirst, nocturia/nocturnal enuresis, low blood pressure, constipation, carpopedal spasms, or tetanic episodes triggered by hypomagnesemia. Treatment of hypomagnesemia is difficult. In GS patients with hypomagnesemia, magnesium supplementation should be considered first, which can accelerate potassium repletion to reduce the risk of tetany and other complications (29, 30). Oral magnesium supplements are appropriate to correct hypomagnesemia, which deteriorates to hypokalemia and makes it difficult to supply potassium. Although all types of magnesium salts are effective (31), their bioavailability is highly variable, resulting in osmotic diarrhea at high doses (32). For patients with acute and severe complications of hypomagnesemia (such as tetany, arrhythmia) or digestive intolerance to oral magnesium supplements, intravenous infusion of magnesium should be used (3). Furthermore, a series of drugs should be avoided for patients with GS, including drugs that slow down the sinus rhythm or influence the QT interval, drugs that can potentially exacerbate hypomagnesemia, and acetazolamide.

Above all, GS patients often present with muscle weakness and fatigue caused by hypokalemia and hypomagnesemia, which contribute to a significant reduction in quality of life. Therefore, early diagnosis of GS is necessary in young children to reduce the possibility of growth retardation, tetany, and seizures. NGS such as whole-exome or whole-genome sequencing provides a practical tool for the early diagnosis and improvement of the prognosis of GS. Importantly, further whole-genome sequencing is expected to reveal more variants in *SLC123A* among GS patients with single heterozygous mutations.

## Data Availability Statement

The datasets presented in this study can be found in online repositories. The names of the repository/repositories and accession number(s) can be found at: NCBI, SRA repository with accession number SUB7270931.

## Ethics Statement

The studies involving human participants were reviewed and approved by The Ethics Committee of the Children Hospital of Zhejiang University School of Medicine. Written informed consent to participate in this study was provided by the participants' legal guardian/next of kin.

## Author Contributions

LZ, KH, and SW contributed to patient service and data collection. CF, GD, and JM contributed to study conception and design. HF, JW, HS, and ZL collected the data. JC, YB, and SW contributed to data analysis and interpretation. All authors participated in drafting of the article or key modifications of important content and approved the final version to be published.

## Conflict of Interest

The authors declare that the research was conducted in the absence of any commercial or financial relationships that could be construed as a potential conflict of interest.
